# Observations upon Inveterate Ulcers

**Published:** 1799-11

**Authors:** J. Vage


### *Obſervations upon Inveterate Ulcers; by* J. VAGE, *M. D.*

### *To the Editors of the Medical and Phyſical Journal.*

GENTLEMEN,

THE ingenious exertions of ſome of your correſpondents for the cure of old ulcers, and particularly in the lower extremities, induces me to contribute my mite to this laudable intention. A great number of caſes of this deſcription has been, for ſeveral years paſt, under my care; and the inveteracy of ſome of them cauſed me to be ſomewhat experimental in their treatment. The reſult of my trials, both in reſpect of theory and practice, I ſhall communicate as briefly as I can for your uſeful publication. In this, however, I muſt beg leave to ſpeak agreeably to the doctrines of the old ſchool; for, though the preſent rage of applying the modern chemical diſcoveries to the practice of phyſic is highly commendable, yet as a warm controverſy ſubſiſts between men of eminent abilities, not only about all the applications which have been made of theſe diſcoveries, but even about the diſcoveries themſelves, it warrants me to decline them, until indubitable facts, and cool impartial teſtimonies, ſhall have decided the buſineſs on one ſide or other.

Ulcers

Ulcers have been long diftinguifhed into benign and malignant, in proportion as they have yielded to or refifted the eftablifhed methods of practice; for, indeed, whatever advancements medical knowledge may have received, in many points this branch of it appears to have fo ftubbornly retained its difficulties, that even at this time the ableft practitioners are frequently obliged to plead the defectivenefs of the art. Formerly, the obftinacy of ulcers were acknowledged to be fo great, as to be attributed to witchcraft. But when the progrefs of natural knowledge had diffipated the clouds of fuperftition, another afylum was founded for incapacity; and ulcers, whenever they baffled the furgeon's fkill, were fuppofed to belong to difeafes which are commonly efteemed incurable, and they were then denominated of a fcrofulous, or cancerous nature, and the like. Many authors, with more reafon and fcientific latitude on their fide, imagined that the malignity of fuch cafes always proceeded from fome bad habit of body, fome depravation in the fluids and folids, and I was eafily led to efpoufe this tenet; but obferving ftill that many ulcers, which could not with any propriety be reckoned either fcrofulous or cancerous, not only eluded the ftricteft regimen, and a perfeverance in the moft approved alterative courfes of medicine, but were even aggravated in their condition, I began to fufpect this doctrine too, and that inftead of any general conftitutional fource, the caufe of their malignity and obftinacy was chiefly of a local nature. A comparative view of a foul ulcer, with one in a healing ftate, feemed to correfpond fo exactly with this opinion, that, extraordinary as it appeared, I was encouraged to adopt it. In an inveterate ulcer, it is obfervable that the external parts about it, and, of courfe, the parts beneath its furface, are in a morbid ftate; and as the humours which conftitute the difcharge muft pafs through thefe parts, it is reafonable to fuppofe that they contract a degree of acrimony, or fome quality inimical to the furface of the fore, and, confequently, become corrofive and painful. Upon any temporary ceffation of this pain and corrofion, which is often procured by palliative applications, either a gloffy, fpongy, inorganized flefh fprings up, or little callous granulations, at the bottom and fides of the ulcer. In a healing one, every thing is the reverfe of this; the contiguous parts are in a found condition, and have a natural complexion; the granulations, which gradually proceed to incarn the fore, have a healthy appearance, and are properly organized with veffels and nerves for a circulation in the future cicatrix; the difcharge is a well-digefted pus, which, inftead of exciting pain, is nature's congenial balfam to fheath and cherifh the tender germinations.

But

But what chiefly folicited my attention was, that all thefe appearances in malignant ulcers, their acrimony, the fanioufnefs of the difcharge, the corrofion, the fpongy flefh, the callous granulations, &c. were always in proportion to the extent of the morbid circumjacent parts, and the extent itfelf, the product of habitual virulence. A fickly complexion, and a vitiated conftitution, indeed, conftantly attend ulcers of any confiderable ftanding or magnitude; and hence, probably, arofe the opinion that their malignity originated from that fource. It is, however, pretty certain, that this depravation of the fluids and folids, though it may in its turn increafe the obftinacy of ulcers, is yet, in general, the effect and not the caufe of them, from an abforption of the morbid humours generated in and about them. If this is the cafe, there is much reafon for thinking that artificial fores, if long continued, whatever advantage they afford at firft, fuch as iffues, fetons, perpetual blifters, become hurtful to the conftitution. And I am not without reafons for believing, that even the cicatrices of old ulcers long circulate and fecrete humours injurious in fome degree to the animal œconomy.

But to bring as near as poffible to a proof, and to afcertain with fome certainty what effects a reputed bad habit of body, or fuch a one as accompanies old ulcers, has in producing the acrimony of their difcharge, I caufed artificial fores to be made with cauftics, in patients afflicted with fuch ulcers, and in fimilar parts of the body; for it appeared a fair conclufion, that where a putrid efchar was to be feparated and digefted off by an inflammation and a generation of found flefh, the general depravity of the folids and fluids, if it was powerful enough to keep up the virulence of the old ulcer, would fhew a fimilar difpofition in and retard the cure of the new one. No fuch thing happened; but in many patients felected for the purpofe, at different times, the efchars digefted off, and the wounds healed as kindly, as they would have done in people without any previous ailment. What other inference, then, could be drawn from this, but that the malignity of old ulcers chiefly arifes from the morbid circumjacent parts, and that the deftruction of thefe parts, or, in other words, a reduction of fuch ulcers to the ftate of a frefh wound, would infallibly bring them to a healing condition? I made no fcruple, therefore, to put this idea in execution, and always found where it could be perfectly done, it never failed of fuccefs. What are all the common modes of practice, in fevere cafes, but mere palliatives? It is eafy to correct the difcharge; farinaceous and vegetable cataplafms in a ftate of fermentation, feveral antifeptic liniments, and fpirituous camphorated acetated dreffings, will do it, and procure a tema temporary relief; but that ſtate is followed by nothing more than either the generation of that gloſſy ſpongy fleſh already mentioned, or the callous knobby granulations, which are only as bad or worſe variations of the complaint.

Long before I had adopted this way of thinking and practice, I knew that cauſtics would effectually ſtop and quickly heal ſome venereal chancres; but I remarked, at the ſame time, that ſome were ſo virulent as to reſiſt them, which I was then at a loſs to account for; but I now know to a certainty, that the failure aroſe from too ſuperficial an application of them. The cauſtics which were then uſed, were the lunar, and a ſolution of argent viv. in the nitrous acid; and I, for ſome time, imagined, that the good effects were peculiar to theſe; but the ſtrong cauſtic alkali anſwers better: it occaſions leſs pain, as it inſtantly deſtroys the ſenſation of the parts, and, therefore, the ſtronger it is the better, if it is uſed with judgment.

